# An analysis of marketing authorisation applications via the mutual recognition and decentralised procedures in Europe

**DOI:** 10.1007/s00228-015-1904-1

**Published:** 2015-07-25

**Authors:** Hans C. Ebbers, Joris Langedijk, Jacoline C. Bouvy, Jarno Hoekman, Wouter P. C. Boon, Jean Philippe de Jong, Marie L. De Bruin

**Affiliations:** Division of Pharmaceutics, Faculty of Science, Utrecht Institute for Pharmaceutical Sciences, Utrecht University, PO box 80082, 3508 TB Utrecht, The Netherlands; Division of Pharmacoepidemiology and Clinical Pharmacology, Faculty of Science, Utrecht Institute for Pharmaceutical Sciences, Utrecht University, PO box 80082, 3508 TB Utrecht, The Netherlands; Medicines Evaluation Board (CBG-MEB), Utrecht, The Netherlands; Innovation Studies Group, Faculty of Geosciences, Utrecht University, Utrecht, The Netherlands; Exon Consultancy, Amsterdam, The Netherlands

**Keywords:** MRP/DCP procedure, CMDh referrals, Marketing authorisation (MA), Regulatory science, Pharmaceutical regulation

## Abstract

**Purpose:**

The aim of this study is to provide a comprehensive overview of the outcomes of marketing authorisation applications via the mutual recognition and decentralised procedures (MRP/DCP) and assess determinants of licensing failure during CMDh referral procedures.

**Methods:**

All MRP/DCP procedures to the Co-ordination group for Mutual recognition and Decentralised procedures–human (CMDh) during the period from January 2006 to December 2013 were analysed. Reasons for starting referral procedures were scored. In addition, a survey under pharmaceutical companies was performed to estimate the frequency of licensing failure prior to CMDh referrals.

**Results:**

During the study period, 10392 MRP/DCP procedures were finalized. Three hundred seventy-seven (3.6 %) resulted in a referral procedure, of which 70 (19 %) resulted in licensing failure, defined as refusal or withdrawal of the application. The frequency of CMDh referrals decreased from 14.5 % in 2006 to 1.6 % in 2013. Of all referrals, 272 (72 %) were resolved through consensus within the CMDh, the remaining 105 (28 %) were resolved at the level of the CHMP. Most referrals were started because of objections raised about the clinical development program. Study design issues and objections about the demonstration of equivalence were most likely to result in licensing failure. An estimated 11 % of all MRP/DCP procedures resulted in licensing failure prior to CMDh referral.

**Conclusion:**

Whereas the absolute number of MRP/DCP procedures resulting in a referral has reduced substantially over the past years, no specific time trend could be observed regarding the frequency of referrals resulting in licensing failure. Increased knowledge at the level of companies and regulators has reduced the frequency of late-stage failure of marketing applications via the MRP/DCP.

**Electronic supplementary material:**

The online version of this article (doi:10.1007/s00228-015-1904-1) contains supplementary material, which is available to authorized users.

## Introduction

Several regulatory pathways exist to authorise medicines in the European Union (EU). The centralised procedure was introduced in European legislation in 1993 and came into operation in 1995 [1, 2]. It results in a single marketing authorisation (MA) that is valid throughout the EU. The centralised procedure is mandatory for marketing authorisation applications (MAAs) of new active substances for the treatment of HIV/AIDS, cancer, diabetes, neurodegenerative diseases, auto-immune and other immune dysfunctions, and viral diseases, all biologicals, advanced therapies, and orphan products. Applications for multiple Member States for products that do not fall within the mandatory scope of the centralised procedure must follow the mutual recognition procedure (MRP) or the decentralised procedure (DCP). In terms of volume, MRP and DCP procedures outnumber the centralised procedure and considerable resources are spent by both MA holders and national competent authorities on MAAs via the MRP/DCP procedures. When MAAs result in licensing failure—defined as those procedures that did not result in a MA—this leads to wasted resources, especially if this concerns preventable, late-stage failures. Whereas reasons for licensing failure for products authorised via the centralised procedure has received considerable attention, little is known about MAAs via the MRP/DCP procedure [3, 4].

Since January 1, 1998, the MRP is mandatory for any product that is to be marketed in multiple Member States, when a MA exists anywhere in the EU [5]. During the MRP, an applicant informs the Reference Member State (RMS) that it aims to market a product in multiple countries and requests these other countries, the so-called concerned member states (CMSs), to recognise the MA granted by the RMS. The RMS circulates the assessment report, including the approved summary of product characteristics (SmPC), labelling and package leaflet. If the CMSs agree with the assessment of the RMS, they should recognise the decision within 90 days after receipt of these documents by granting a national MA (Fig. S1) [6].

The DCP was introduced into European legislation in 2004 and should be followed when a MA is applied for in multiple Member States at once [7]. Like the MRP, the DCP is also based on recognition of a first assessment performed by a RMS, but there is no preexisting MA. For both MRP and DCP procedures, a positive outcome will result in harmonised national MAs, granted by the respective national competent authorities. After a positive outcome of the MRP/DCP procedure (i.e. all CMSs agree to grant the MA), the procedure is closed and a national MA should be granted within 30 days, provided that well-translated documents are provided within 5 days after closing the procedure.

Member States can refuse to recognise the assessment of the RMS, but only on grounds of a ‘potential serious risk to public health’ (PSRPH). A PSRPH is defined as ‘a situation where there is a significant probability that a serious hazard resulting from a human medicinal product in the context of its proposed use will affect public health’ [8]. Despite the development of guidance, uncertainty remains about what qualifies as a PSRPH [9]. If disagreement on the PSRPH cannot be resolved by the RMS and the CMSs, the issue is referred to the Co-ordination group for mutual recognition and decentralised procedures–human (CMDh), through a so-called Article 29(1) procedure. The CMDh works by achieving consensus between the Member States. If it does not achieve consensus to approve or refuse the MAA within 60 days, the case is referred to the Committee for Medicinal Products for Human Use (CHMP) through an Article 29(4) procedure who will adopt an opinion that will result in a binding decision from the European Commission [10].

Limited data are currently available on the outcomes of MAAs via the MRP/DCP procedure. Furthermore, data on licensing failure prior to MRP/DCP procedures are not available from publicly accessible sources. Therefore, the current study aims to assess the efficiency of the MRP/DCP procedure by providing a comprehensive overview of the outcomes with these regulatory pathways. To do so, we have investigated frequencies and determinants for CMDh referral procedures, as well as reasons for licensing failure during the MRP/DCP. Three objectives were formulated. The first objective was to determine the frequency of CMDh referrals. The second objective was to assess the association of objections raised as PSRPH and other determinants with licensing failure during CMDh referrals. The third objective of this study was to determine the frequency of licensing failure of MAAs via the MRP/DCP prior to the initiation of a CMDh referral procedure.

## Methods

Data were obtained from different sources. The total number of MRP/DCP procedures finalised between January 2006 to December 2013 and all data relating to Article 29(1) procedures, including procedure type (i.e. DCP or MRP), legal basis (see Table S1) and prescription status, were obtained from statistics and reports available from the CMDh website [11]. Additional data on individual products, including pharmaceutical form and legal status were retrieved from public assessment reports that were obtained via the Mutual Recognition Product Index [12]. Article 29(4) commission decision reports were obtained from the European Commission pharmaceuticals community register [13]. Our analysis was limited to initial MAAs; renewal procedures and type II variations were excluded.

A scoring system was developed to categorise objections raised during the CMDh procedure (see Table S2 of the Supplementary information). Two researchers (HE and JL) independently scored the objections; disagreement was resolved by consensus. Multiple objections were scored as ‘Multiple objections from different categories’, unless the issues concerned the same category. Licensing failure was defined as a MAA procedure that did not result in a MA and included negative results at the level of the CMDh, a negative European Commission decisions, or withdrawals by the applicant.

MAAs via the MRP/DCP may also result in licensing failure prior to the start of a CMDh referral. When an MAA is withdrawn before day 90 of the MRP (including the preexisting MAs) or day 120 of the DCP procedure, the information will not be reported on the CMDh website and was thus not available for our study. Therefore, a survey was conducted under 58 member companies of the European Federation of Pharmaceutical Industries and Associations (EFPIA) and the Association of the European Self-Medication Industry (AESGP) to estimate the frequency of licensing failure during the early phase of the MRP/DCP procedure. The European Generic Association (EGA) declined the invitation to participate in the survey. The survey also included questions on the consequences of PSRPHs raised during the MRP/DCP.

All data were entered into a database, and descriptive statistics were obtained using IBM SPSS statistics version 20.0.0 (IBM Corporation, 2011). Significance for numerical variables was tested using Mann-Whitney *U* test (two-sided α < 5 %).

## Results

### Frequency of referral procedures

A total of 10,392 MRP/DCP procedures were finalised during the study period, 2822 MRP and 7570 DCP procedures (Table 1). Generic applications accounted for 78 % of the procedures and hybrid procedures for 10 %. Full dossiers were provided for 6 % of the applications, bibliographic applications accounted for 4 % and the remaining 2 % concerned other applications (see Table S1). Most MAAs concerned products that were authorised as prescription-only in the RMS.

**Table 1:** Total number of marketing authorisation applications and CMDh referrals

		Total, *n*	Referrals, *n*	Percent	Risk ratio (95 % CI)
Procedure type	DCP	7570	135	1.8 %	Ref
	MRP	2822	242	8.6 %	4.8 (3.9–5.9)
Period^a^	2010–2013	6140	70	1.1 %	Ref
	2006–2009	4245	307	7.2 %	6.3 (4.9–8.2)
Prescription status (in RMS)	Prescription only	9890	356	3.6 %	Ref
	Non-prescription	502	21	4.2 %	1.2 (0.8–1.8)
Legal basis*	Art. 10.1—Generic	8120	248	3.1 %	Ref
	Art. 10.3—Hybrid	1010	29	2.9 %	0.9 (0.6–1.4)
	Art. 8.3—Full dossier	600	63	10.5 %	3.4 (2.6–4.5)
	Art. 10a—Well-established use (bibliographic)	439	29	6.6 %	2.2 (1.5–3.1)
	Art 10b—Fixed combination	147	8	5.4 %	1.8 (0.9–3.5)
	Other	56	0	0 %	NA

^a^Total numbers differ from procedure type and prescription status categories due to missing data

**Fig. 1:**
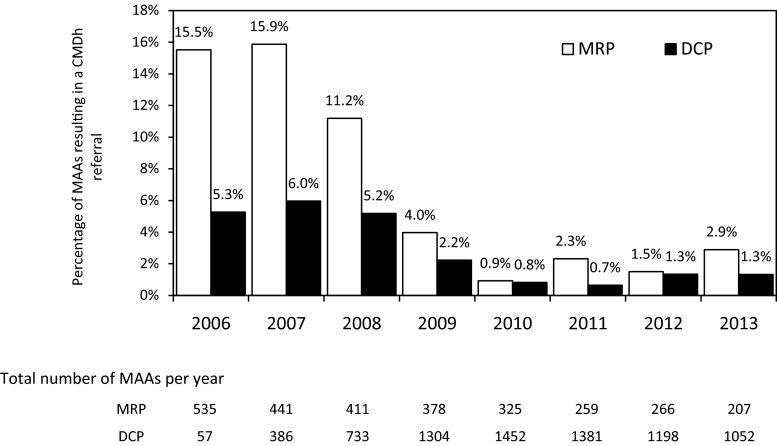
Percentage of procedures resulting in CMDh referral per year

While MRP procedures predominated in 2006 and 2007, from 2008, DCP procedures accounted for the majority of the MAAs. During the study period, 377 (3.6 %) CMDh referral procedures were started. During the first years after the introduction of the DCP, more procedures resulted in a referral, compared to more recent years (Fig. 1). For the combined MRP/DCP procedures, the frequency of CMDh referrals declined from 14.5 % in 2006 to 1.6 % in 2013. MRP procedures were nearly five times more likely to result in a referral than DCP procedures (Table 1). MAAs based on a full dossier and on bibliographic data were more likely to result in a referral compared to generic applications. No difference in the frequency of CMDh referrals was observed for prescription versus nonprescription medicines.

### Assessment of determinants of licensing failure during the CMDh referrals

**Fig. 2:**
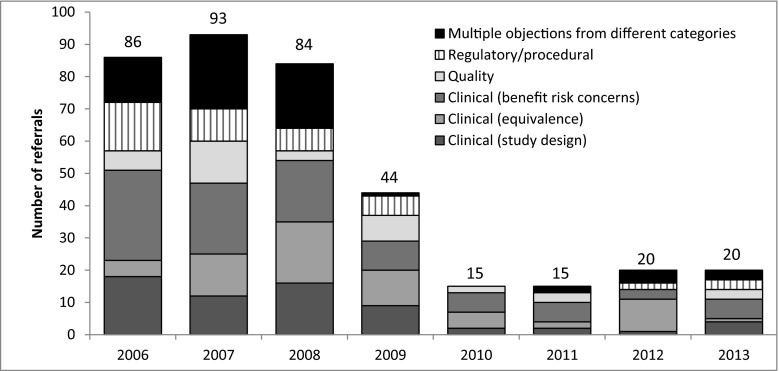
Main categories of ‘potential serious risk to public health’ objections per year. A detailed overview of the category of objection by subcategory and licensing outcome is provided in supplementary Table S3

Of the 377 CMDh referrals, consensus was found within the CMDh for 272 (72 %) referrals, leading to a positive opinion for 239 (63 %) MAAs and licensing failure for 33 (9 %) MAAs. Article 29(4) procedures (CHMP arbitrations) were started for 105 (28 %) MAAs. Of these, 37 (10 %) ended in a refusal and 68 (18 %) resulted in a positive recommendation from the CHMP. So, overall, 70 (19 %) MAAs resulted in a licensing failure. Two illustrative cases that were referred to the CMDh are presented in supplementary Box 1. The majority of PSRPH leading to a CMDh referral procedure were related to the clinical phase (Table 2). PSRPHs concerning the main category *benefit*-*risk concerns* accounted for most CMDh referrals. PSRPHs related to the design of the clinical studies and the demonstration of therapeutic equivalence and bioequivalence were more likely to result in a licensing failure during the referral procedure, than referrals started because of benefit/risk concerns, quality or regulatory/procedural objections. For 88 referrals, multiple objections from different categories were raised (see Table S4 for more detailed information on the combinations). The number of CMDh referrals was small, especially in the second half of the study period. No time trends could be observed in terms of relative frequency of the categories of PSRPH leading to CMDh referral. (Fig. 2 and supplementary Table S3).

**Table 2:** Categories of ‘potential serious risk to public health’ objections raised leading to CMDh referrals and licensing failure during CMDh referrals

Main category Subcategories^a^	Total, *n*	Licensing failure, *n*	Percent	Risk ratio (95 % CI)
Clinical (study design issues)	64	21	33 %	Ref
Clinical (equivalence)	64	21	33 %	1.0 (0.6–1.6)
Bioequivalence/therapeutic equivalence *not demonstrated*	39	15	38 %	1.2 (0.7–2.0)
Bioequivalence/therapeutic equivalence *not investigated in subgroup*	25	6	24 %	0.7 (0.3–1.6)
Clinical (benefit risk concerns)	83	8	10 %	0.3 (0.1–0.6)
Insufficient data to support B/R in claimed indications	34	7	21 %	0.6 (0.3–1.3)
Safety concerns	19	0	0 %	NA
Overall benefit/risk negative	18	1	6 %	0.2 (0.0–1.1)
Posology concerns	12	0	0 %	NA
Quality	38	3	8 %	0.2 (0.1–0.8)
Concerns on quality or manufacturing parameters	35	3	9 %	0.3 (0.1–0.8)
Packaging concerns/medication errors	3	0	0 %	NA
Regulatory/procedural	40	2	5 %	0.2 (0.0–0.6)
Concerns about SmPC wording	30	1	3 %	0.1 (0.0–0.7)
Administrative concerns (including patient leaflet /SmPC issues)	10	1	10 %	0.3 (0.0–2.0)
Multiple objections from different categories	88	15	17 %	0.5 (0.3–0.9)
Overall	377	70	19 %	

^a^For a detailed description of the categories, see supplementary information Table S2

No association was observed between licensing failure and active substance type, administration route, prescription status or MRP vs. DCP application during the referral procedure (Table 3). Referrals of MAAs based on a full dossier (Article 8.3) were less likely to result in licensing failure. Cardiovascular products and nervous system products were the two product classes most frequently included in CMDh referrals. Antineoplastic and immunomodulating agents and genitourinary system and sex hormones were less likely to result in licensing failure when compared to cardiovascular agents. The Netherlands, Germany, Denmark, the UK and Sweden together acted as RMS for 78 % of all referrals. Procedures in which the Netherlands or Sweden were RMS, were less likely to result in licensing failure, whereas procedures where Denmark was the RMS more often resulted in licensing failure, when compared to all other Member States. Per procedure, a median of 8 (IQR 4–12) CMSs were involved. Procedures that resulted in licensing failure involved fewer CMSs (5.5; IQR 1–9) than procedures with a positive outcome (8; IQR 4–23; *p* < 0.001). This difference remained when we limited our analysis to only MRP, or only DCP procedures. No specific time trends were observed for the frequency of licensing failure.

**Table 3:** Other determinants of licensing failure during CMDh procedures

Category	Subcategory	Total	Licensing failure	Percent	RR (95 % CI)
Procedure type	DCP	135	29	21.5 %	Ref
	MRP	242	41	16.9 %	0.8 (0.5–1.2)
Period	2006	86	14	16.3 %	Ref
	2007	93	23	24.7 %	1.6 (0.9–2.8)
	2008	84	11	13.1 %	0.8 (0.4–1.7)
	2009	44	5	11.4 %	0.7 (0.3–1.8)
	2010	15	5	33.3 %	2.1 (0.9–4.9)
	2011	15	4	26.7 %	1.7 (0.6–4.4)
	2012	20	2	10.0 %	0.6 (0.2–2.5)
	2013	20	6	30.0 %	1.9 (0.8–4.2)
Prescription status (in RMS)	Prescription only	356	65	18.3 %	Ref
	Non-prescription	21	5	23.8 %	1.3 (0.6–2.9)
Legal basis	Art. 10.1—Generic	248	50	20.2 %	Ref
	Art. 8.3—Full dossier	63	4	6.3 %	0.3 (0.1–0.8)
	Art. 10.3—Hybrid	29	8	27.6 %	1.4 (0.7–2.6)
	Art. 10a—Well-established use (Bibliographic)	29	8	27.6 %	1.4 (0.7–2.6)
	Art. 10b—Fixed combination	8	0	0.0 %	NA
Active substance type	Small molecules	361	68	18.8 %	Ref
	Biologicals^a^	16	2	12.5 %	0.7 (0.2–2.5)
Route of administration	Oral	264	48	18.2 %	Ref
	Injectables	40	4	10.0 %	0.6 (0.2–1.4)
	Other systemic	30	6	20.0 %	1.8 (0.8–3.7)
	Inhaled	16	5	31.3 %	1.7 (0.8–3.7)
	Topical	16	3	18.8 %	1.0 (0.4–2.0)
	Other	11	4	36.4 %	2.0 (0.9–4.6)
ATC level	C—Cardiovascular system	88	23	26.1 %	Ref
	N—Nervous system	76	17	22.4 %	0.9 (0.5–1.5)
	J—Anti-infectives for systemic use	38	4	10.5 %	0.4 (0.1–1.1)
	A—Alimentary tract and metabolism	34	4	11.8 %	0.5 (0.2–1.2)
	L—Antineoplastic and immunomodulating agents	30	1	3.3 %	0.1 (0.0–0.9)
	R—Respiratory system	30	9	30.0 %	1.1 (0.6–2.2)
	G—Genitourinary system and sex hormones	29	1	3.4 %	0.1 (0.0–0.9)
	M—Musculoskeletal system	21	3	14.3 %	0.5 (0.2–1.7)
	Other	28	5	17.9 %	0.7 (0.3–1.6)
	Unknown^b^	3	3	100 %	
RMS	Other	86	22	25.6 %	Ref
	The Netherlands	81	4	4.9 %	0.2 (0.1–0.5)
	Germany	68	9	13.2 %	0.5 (0.3–1.1)
	Denmark	54	29	53.7 %	2.1 (1.4–3.2)
	UK	51	6	11.8 %	0.5 (0.2–1.1)
	Sweden	37	0	0.0 %	NA

^a^Teicoplanin included in the biologics group

^b^All Article 8.3 procedures (‘full dossiers’) that did not receive marketing authorisation (excluded from analysis)

### Licensing failure prior to initiating a CMDh referral

In total, 16 of the 58 (28 %) invited companies returned the survey. Of these, four companies provided two surveys from different departments within the same company, e.g., consumer health care and innovative medicines, or consumer health care and generics. This resulted in 20 completed individual surveys, reporting a total of 208 MRP/DCP procedures (Table 4). Out of all MRP/DCP procedures, 174 (84 %) ended in a MA, whereas 11 % resulted in licensing failure at the level of the RMS (i.e., were refused or withdrawn) prior to CMDh referral, and 10 (5 %) procedures were referred to the CMDh. For 20 (10 %) of the procedures, the applicant withdrew the application in one or more Member States. The majority of the withdrawals were reported to occur for reasons other than safety concerns. Five respondents (25 %) indicated that their company had withdrawn MAAs (and MAs) in response to safety concerns at least once. Of all the respondents, 21 % reported that their company had decided not to market a product in one or more Member States because of restrictions on the use of the product introduced during the MRP/DCP procedure at least once.

**Table 4:** Survey results on marketing authorisation applications using MRP/DCP

	Total	Procedures resulting in a MA	Procedures resulting in licensing failure	CMDh referral
Completed in all Member States	174 (84 %)	156 (75 %)	9 (4 %)	9 (4 %)
Withdrawn ≥ 1 Member States^a^	20 (10 %)	19 (9 %)	0	1 (<1 %)
Withdrawn in *all* Member States prior to CMDh referral^b^	14 (7 %)	0	14 (7 %)	0
Total number of procedures	208 (100 %)	175 (84 %)	23 (11 %)	10 (5 %)

^a^Outcome in remaining Member States

^b^Including the existing marketing authorisation in the RMS

## Discussion

We have provided a comprehensive overview of MAAs via the MRP/DCP. We found that only a limited number of applications are referred to CMDh, and the majority of these referrals resulted in a MA. PSRPH objections that related to the design of the clinical studies and the demonstration of therapeutic equivalence and bioequivalence were most likely to result in a licensing failure, whereas discussion on quality or regulatory concerns rarely resulted in a licensing failure during the procedure. Some factors, including procedure type, legal basis and timing of the procedure were associated with the frequency of triggering a CMDh referral, but not with a higher rate of negative outcomes once the referral was initiated. Overall, these data show that the frequency of late-stage licensing failure of MRP/DCP procedures, i.e., licensing failure after referral, has decreased substantially.

Care must be taken when interpreting outcomes of regulatory procedures. We defined licensing failure as a withdrawal or refusal, but this does not mean that the procedure failed. On the contrary, it may imply that the DCP/MRP functions as expected and prevented (potential) untoward outcomes resulting from subpar products reaching patients. Moreover, our study focused on *overall* licensing failure, meaning that we did not take into account that for some products, the authorised indications and/or patient populations may have been restricted at the end of the MRP/DCP procedure. Respondents to the survey reported that this had on occasion resulted in decisions not to market a product. However, we did not systematically investigate the underlying reasons for those restrictions. This may be a topic for further study.

The frequency of MAAs that resulted in a CMDh referral decreased substantially over the years, indicating that regulatory learning takes place. Increased experience in the use of this pathway may have resulted in improved MAAs filed by companies, but also in earlier withdrawal of applications that are likely to result in a referral. Companies may also adapt their filing strategies to anticipate regulatory concerns and file in selected Member States. For regulators, regulatory learning means that they may have become better in finding consensus about MAAs in earlier phases of the application, but also the development of guidance on what are considered PSRPHs may reduce disagreements between different Member States [9]. Furthermore, an ever-increasing body of information about outcomes of referral and arbitration procedures will provide more clarity on the interpretation of PSRPHs and prevent referrals. Work within the CMDh is ongoing to improve the harmonised interpretation of existing guidance [14]. Moreover, ongoing harmonisation efforts of SmPCs of products for which Member States have adopted different decisions over the years (resulting in different authorised indications, contraindications or posology) will continue to reduce sources of disagreement [15].

Our data clearly show that MRP procedures result in CMDh referrals more frequently than DCP procedures. A possible explanation for this finding is that the RMS is more reluctant to accept changes to the existing SmPC, than in the situation of a DCP, where there is no preexisting MA. Moreover, given the fact that DCPs do not have preexisting MAs, companies may withdraw an MAA more easily in response to objections raised during the assessment procedure, in order to resubmit with different claims, or in different member states.

Objections raised on the design and outcome of clinical studies were most likely to lead to licensing failure. Often, these objections related to bioequivalence parameters that were outside predefined borders, even when the studies were adequately designed. These cases may be the result of unforeseen differences in the product characteristics or due to chance findings, which may be challenging to prevent. On the other hand, a considerable amount of referrals were due to causes that may have been prevented by the applicant through early communication with the competent authorities, such as the choice of reference product or dosage strength. Consequently, careful planning of clinical studies and consideration of existing guidelines could further reduce the frequency of referrals.

We found that procedures resulting in licensing failure involved fewer CMSs than those that resulted in a MA. This seems counterintuitive, as more CMSs would give rise to more opportunity for disagreement. A possible explanation may be that applicants anticipate objections and file in strategically selected Member States. For example, it has been recognised that the MRP/DCP is underutilised by the non-prescription sector, because of different approaches towards self-medication in the member states [16]. While we did not observe a higher frequency of licensing failure for non-prescription medicines compared to prescription medicines, companies may anticipate concerns during the procedure and run multiple procedures for the same product, leading to fewer referrals.

We found that five RMSs accounted for 78 % of all referrals. However, these five countries also acted as RMSs for 69 % of all existing MAs included in the Mutual Recognition Product Index (Table S4) [12]. ATC classes of authorised products were also distributed unevenly over the RMSs (data not shown), which may also account for some of the observed variation in the licensing failure frequency seen in our study. It may be of interest to further investigate the underlying reasons for the observed differences in frequency of licensing failures between RMSs.

Data from our survey suggest that 16 % of all MAAs via the MRP/DCP procedures were withdrawn in one or all Member States at some point. This suggests that companies anticipate that objections will be raised and take mitigating measures.

### Strengths and limitations

Our study was the first to provide a comprehensive overview of MAAs via the mutual recognition and decentralised procedures. An important limitation of our study is that for the MAAs which did not result in a referral various attributes were only available on an aggregated level, such as legal basis, prescription status and procedure. While these did not show major differences over the years, we were unable to perform multivariate analyses to identify explanatory variables for changes in the frequency of referrals over time. Other variables, including RMS, ATC class, and route of administration, were unavailable altogether.

Multiple data sources were required to obtain a full picture on the outcomes of MRP/DCP procedures. While it may be preferable to use a single data source, the use of multiple data sources allowed us to validate our findings. For example, it may not be possible to extrapolate our survey results to all users of the MRP/DCP procedures, as our sample included only a few generic companies. Nevertheless, in our survey, 10 out of 208 procedures (4.8 %) resulted in a CMDh referral. This is comparable to the number of referrals included in the CMDh database (377/10,392 = 3.6 %), providing some reassurance with respect to the representativeness of the survey sample. The data of the current study are also in accordance with data from another study that investigated licensing failure of DCP applications filed in the Netherlands and found that 9.8 % resulted in licensing failure (Langedijk et al., manuscript in preparation). This is in the same range as the 7.9 % observed in our survey (where 7 % of the applications were withdrawn prior to CMDh referral and an estimated 0.9 % failed during CMDh referral).

## Conclusion

A limited number of MRP/DCP procedures in our study ended in a CMDh referral, and the frequency of referrals has decreased substantially in recent years, indicating that companies and regulators have learnt to prevent late-stage failures of MAAs via the MRP/DCP. Ever-increasing experience in using the MRP/DCP results in a growing body of information about past referral outcomes that may facilitate the development of strategies to prevent licensing failure late in the procedure. Ongoing harmonisation activities on the side of regulatory authorities will likely lead to a further reduction of licensing failure during the MRP/DCP procedure.

## Electronic supplementary material

ESM 1 (DOCX 65 kb)
