# Supplementary material for: An analysis of marketing authorisation applications via the mutual recognition and decentralised procedures in Europe
Source: Eur J Clin Pharmacol. 2015 Jul 25;71(10):1237–44. doi: 10.1007/s00228-015-1904-1 (PMC4564446; doi:10.1007/s00228-015-1904-1)
Supplement: Supplementary file 1 — (DOCX 65 kb) [file 228_2015_1904_MOESM1_ESM.docx]

## Supplementary information 24/06/2015

**Figure 1**. Flowchart of MRP/DCP procedures.

Day 105

Clock stop to allow applicant to supplement the dossier and answer questions.

Day 70

CMSs send their comments differentiating between PSRPH and points for clarification.

Day 150 (CHMP)

Article 29(4) procedure resulting in an opinion from the CHMP and a binding decision at the European Commission.

Day 90 (CMDh)

Article 29(1) procedure is started.

Day 270 (CHMP)

Article 29(4) procedure resulting in an opinion from the CHMP and a binding decision at the European Commission.

Day 210 (CMDh)

Article 29(1) procedure is started.

Day 180

A break-out session including the applicant may be organised to resolve differences. If no consensus, CMSs inform RMS, applicant and CMDh of final negative position. RMS forwards AR, proposed SmPC, PL and labelling, including ground for referral to CMDh.

MRP

DCP

Day 60

A break-out session including the applicant may be organised to resolve differences. If no consensus, CMSs inform RMS, applicant and CMDh of final negative position. RMS forwards AR, proposed SmPC, PL and labelling, including ground for referral to CMDh.

Day 0 (start)

RMS sends AR including

SmPC, PL and labelling to CMSs.

CMSs send their comments to the RMS and applicant by day 50. Applicant sends response document at day 60 the latest.

Day 120

RMS sends the DAR, draft SPC, draft labelling and draft PIL to CMSs. If no consensus by day 150 CMSs send final comments to RMS. RMS communicates outstanding issues to applicant who can send additional clarification that is circulated to CMSs.

Day 106

RMS updates PrAR to prepare *Draft* Assessment Report (DAR) SmPC, PL and labelling (no new data is allowed to be submitted after this point).

Day 0 (start)

RMS drafts preliminary Assessment Report (PrAR), SPC, PL and labelling to CMSs and applicant.

Day -14 (pre-procedural step)

Applicant sends dossier to RMS and CMSs.

*Clock stop (6 months maximum)*

Day -14 (pre-procedural step)

Applicant sends dossier to CMSs.

RMS updates AR if necessary.

If consensus is found (or a binding decision is taken at the European Commission) the procedure is closed. If the decision is that the product is approvable, AR, including approved SmPC, PL and labelling and procedure are finalised .

Continue to national step.

Both procedures can be closed if the RMS and CMS reach consensus to approve or refuse the marketing authorisation application. Of note, only procedures that have been positively assessed by the RMS may be referred to the CMDh. Referrals cannot be initiated for applications that are negatively assessed by the RMS, even though one or more CMSs are of the opinion that a MA may be granted.

**Supplementary Box 1: Examples of referrals.**

**Referral that resulted in licensing failure**

The procedures have been referred to the CMD(h) based on a question regarding the requirements to show bioequivalence. The disagreement was regarding whether bioequivalence should be shown for the parent compound losartan or for the active metabolite. The 90% confidence interval for Cmax is within 80-125% for the metabolite (94.8-114.74%), but outside the normal acceptance range for the parent compound losartan (91.26-133.64%). The extraordinarily wide acceptance range for Cmax %CI (70–143%) for the parent compound losartan was not considered acceptable by CMS. An agreement could not be reached and the marketing authorisation applications were withdrawn in RMS and CMS before the CMD(h) meeting.

**Score**: Bioequivalence/therapeutic equivalence not demonstrated.

**Referral that resulted in a marketing authorisation**

There were concerns with regard to the following issues:

1. Bioequivalence was demonstrated on the basis of the metabolite data, and not on the basis of the parent.

2. The pictogram and text in the SmPC and Package Leaflet give the impression that there is a dose recommendation for half a tablet, but there is no such recommendation. This may confuse prescribing physicians and patients.

At the CMD(h) meeting the RMS presented its view and the applicant’s written response was discussed. There was a discussion on whether bioequivalence should be demonstrated only on the active metabolite enalaprilat data, or whether bioequivalence should also be demonstrated on the parent compound enalapril. The applicant made a post-approval commitment to perform an additional single dose fasten bioequivalence study on the parent compound. The SmPC and Package Leaflet were adapted regarding information about the score line. Agreement reached.

**Score**: Combinations of 1. Concerns about the quality of the studies and 2. Administrative concerns (including concerns involving the PL /SmPC).

**Table S1. Legal basis**

| Legal basis |  |  |
| --- | --- | --- |
| Article 8.3 | Full dossier | 1. **Stand-alone application**   An application for marketing authorisation based on:   - pharmaceutical (physico-chemical, biological or microbiological) tests, - preclinical (toxicological and pharmacological) tests, - clinical trials.   For such applications, the relevant published literature also has to be submitted and these scientific publications can be used as supportive data. Mostly used for new active substances.   1. **"Mixed application"**   Applications for marketing authorisation consisting of a combination of reports of limited non-clinical and/or clinical studies carried out by the applicant and of bibliographical references. |
| Article 10.1 | Generic | According to Article 10.1 of Directive 2001/83/EC the applicant is not required to provide the results of pre-clinical tests and clinical trials if he can demonstrate that the medicinal product is a generic medicinal product of a reference medicinal product which has been authorised not less than 8 years in a Member State or in the Union.  A generic medicinal product is defined as a medicinal product that has:   - the same qualitative and quantitative composition in active substance(s) as the reference product, - the same pharmaceutical form as the reference medicinal product, and whose bioequivalence with the reference medicinal product has been demonstrated by appropriate bioavailability studies. |
| Article 10.3 | Hybrid medicinal product | Hybrid applications differ from generic applications in that the results of appropriate pre-clinical tests and clinical trials will be necessary in the following three circumstances:   1. where the strict definition of a ‘generic medicinal product’ is not met; 2. where the bioavailability studies cannot be used to demonstrate bioequivalence; 3. where there are changes in the active substance(s), therapeutic indications, strength, pharmaceutical form or route of administration of the generic product compared to the reference medicinal product.   In such cases the results of tests and trials must be consistent with the data content standards required in the Annex to the Directive 2001/83/EC.  These applications will thus rely in part on the results of pre-clinical tests and clinical trials for a reference product and in part on new data. Some guidance on the appropriate additional studies required is indicated in Annex IV of the Chapter 1 of the Notice to Applicants.  The type of applications mentioned above refer to information that is contained in the dossier of the authorisation of the reference medicinal product, for which a marketing authorisation has been granted in the Union. |
| Article 10a | Well-established medicinal use supported by bibliographic  literature; | For medicinal products the active substance(s) of which has/have a ‘well-established medicinal use’, with recognised efficacy and an acceptable level of safety, the applicant is not required to provide the results of pre-clinical tests or clinical trials if he can demonstrate that the active substances of the medicinal product have been in well-established medicinal use within the Community for at least ten years, with recognised efficacy and an acceptable level of safety in terms of the conditions set out in Annex I, part II of Directive 2001/83/EC. In that event, the test and trial results shall be replaced by appropriate scientific literature. |
| Article 10b | Fixed combination of active substances in a medicinal product | Applications based upon Article 10b shall relate to new medicinal products made of at least two active substances not previously authorised as a fixed combination medicinal product. |

**Table S2.** Scoring categories of reported reasons for CMDh referral

| Main category | Subcategories | Description |
| --- | --- | --- |
| Clinical (equivalence) | Bioequivalence/ therapeutic equivalence *not demonstrated* | BE parameter outside predefined border, endpoint not met for BE/TE studies, post hoc widening of acceptance criteria, exclusion of outliers not supported. |
|  | Bioequivalence/ therapeutic equivalence *not investigated in sub group* | Including dose, fasting/fed condition group, or patient category. Discussions on the acceptability of biowaivers of studies, extrapolation of different dose strengths included in the BE studies. |
| Clinical (study design) | Concerns about the quality of the studies | Study design issues, choice of comparator, choice of test product, GCP compliance, choice of (predefined) endpoints, predefined widening of 90% CI. |
| Clinical (benefit risk concerns) | Insufficient data to support B/R in claimed indications | Including insufficient bibliographic data presented, bibliographic data not enough to support B/R in indications, requests for clinical efficacy studies / therapeutic equivalence studies, claim of well-established use questioned. |
|  | Safety concerns | Concerns raised about adverse events, safety of the product, discussions around the addition/removal of contraindications, special safety warnings and drug interactions. |
|  | Posology concerns | Concerns about posology, including differences in approved posology in different member states, duration of treatment, concomitant therapy, posology in different patient categories. |
|  | Overall B/R negative | Concerns about the overall Benefit/Risk of the product (including cases where claimed efficacy is not sufficiently demonstrated in one or more indications). Including cases where B/R is considered negative. |
| Quality | Concerns on quality or manufacturing parameters | Including stability and safety of excipients, formulation concerns, GMP issues, concerns about the quality/size of the biobatch, impurities, residuals, related substances, etc. |
|  | Packaging concerns / medication errors | Child-resistant packaging, device concerns, device storage orientation, complexity of dispenser, potential for medication errors. |
| Regulatory/procedural | Concerns about SmPC wording | Including differences in approved (contra)indications in different member states for the reference product. |
|  | Administrative concerns (incl. concerns involving the PL /SmPC) | Objections because a product has a different legal status in member states, unclear patient leaflet, unclear SmPC, different SmPCs for different strengths, movement of data to another section of SmPC, providing an RMP, updating patient leaflet, discussions about the legal basis of the application, discussions of acceptability of reference product when the original products was no longer on the market, redefinition of starting material. |

BE= Bioequivalence

GCP= Good clinical practice

GMP= Good Manufacturing Practices

| ***Main category*** | ***Sub category*** | **2006** |  | **2007** |  | **2008** |  | **2009** |  | **2010** |  | **2011** |  | **2012** |  | **2013** |  |
| --- | --- | --- | --- | --- | --- | --- | --- | --- | --- | --- | --- | --- | --- | --- | --- | --- | --- |
|  |  | ***MA*** | ***LF*** | ***MA*** | ***LF*** | ***MA*** | ***LF*** | ***MA*** | ***LF*** | ***MA*** | ***LF*** | ***MA*** | ***LF*** | ***MA*** | ***LF*** | ***MA*** | ***LF*** |
| Clinical (study design) | Concerns about the quality of the studies | 8 | 10 | 6 | 6 | 13 | 3 | 9 | 0 | 2 | 0 | 1 | 1 | 1 | 0 | 3 | 1 |
| Clinical (equivalence) | Bioequivalence/therapeutic equivalence not demonstrated | 2 | 2 | 4 | 2 | 8 | 6 | 6 | 3 | 1 | 2 | 1 | 0 | 1 | 0 | 1 | 0 |
|  | Bioequivalence/therapeutic equivalence not investigated in sub group | 1 | 0 | 3 | 4 | 5 | 0 | 2 | 0 | 0 | 2 | 1 | 0 | 7 | 0 | 0 | 0 |
| Clinical (benefit risk concerns) | Insufficient data to support B/R in claimed indications | 10 | 1 | 6 | 0 | 5 | 1 | 2 | 1 | 2 | 1 | 2 | 1 | 0 | 0 | 0 | 2 |
|  | Safety concerns | 1 | 0 | 7 | 0 | 5 | 0 | 2 | 0 | 1 | 0 | 1 | 0 | 1 | 0 | 1 | 0 |
|  | Posology concerns | 7 | 0 | 3 | 0 | 0 | 0 | 1 | 0 | 0 | 0 | 0 | 0 | 1 | 0 | 0 | 0 |
|  | Overall B/R negative | 2 | 0 | 1 | 0 | 8 | 0 | 2 | 0 | 0 | 0 | 2 | 0 | 0 | 1 | 2 | 0 |
| Quality | Concerns on quality or manufacturing parameters | 6 | 0 | 12 | 0 | 2 | 0 | 8 | 0 | 2 | 0 | 2 | 1 | 0 | 0 | 1 | 2 |
|  | packaging concerns/medication errors | 0 | 0 | 1 | 0 | 1 | 0 | 0 | 0 | 0 | 0 | 0 | 0 | 0 | 0 | 0 | 0 |
| Regulatory/procedural | Concerns about SmPC wording | 11 | 1 | 4 | 0 | 6 | 0 | 6 | 0 | 0 | 0 | 0 | 0 | 2 | 0 | 0 | 0 |
|  | Administrative concerns (incl concerns involving the PL /SmPC) | 3 | 0 | 2 | 1 | 1 | 0 | 0 | 0 | 0 | 0 | 0 | 0 | 0 | 0 | 3 | 0 |
| Combinations of multiple concerns | NA | 21 | 0 | 21 | 10 | 19 | 1 | 1 | 1 | 2 | 0 | 1 | 1 | 5 | 1 | 3 | 1 |
| **Total** |  | **72** | **14** | **70** | **23** | **73** | **11** | **39** | **5** | **10** | **5** | **11** | **4** | **18** | **2** | **14** | **6** |

**Table S3.** Reasons for referral per year and licensing failure

MA= procedures ending marketing authorisation

LF=procedures ending in licensing failure

**Table S4**: Combinations containing at least one of the following

| Category |  | n | % |
| --- | --- | --- | --- |
| Concerns about the quality of the studies |  | 26 | 30% |
| Bioequivalence/therapeutic equivalence *not demonstrated* |  | 13 | 15% |
| Bioequivalence/therapeutic equivalence *not investigated in sub group* |  | 17 | 19% |
| Insufficient data to support B/R in claimed indications |  | 23 | 26% |
| Safety concerns |  | 15 | 17% |
| Posology concerns |  | 29 | 33% |
| Overall B/R negative |  | 8 | 9% |
| Concerns on quality or manufacturing parameters |  | 17 | 19% |
| packaging concerns/medication errors |  | 9 | 10% |
| Concerns about SmPC wording |  | 19 | 22% |
| Administrative concerns (incl. concerns involving the PL /SmPC) |  | 24 | 27% |
| Total number of combinations |  | 88 | 100% |

**Table S5**. Authorised* products via DCP/MRP available in the Mutual Recognition Product Index by Reference Member State

| Country | N | % |
| --- | --- | --- |
| Denmark | 1954 | 17.55% |
| Germany | 1816 | 16.31% |
| United Kingdom | 1643 | 14.76% |
| The Netherlands | 1263 | 11.35% |
| Finland | 1177 | 10.57% |
| Sweden | 956 | 8.59% |
| Portugal | 526 | 4.73% |
| France | 291 | 2.61% |
| Poland | 212 | 1.90% |
| Czech republic | 186 | 1.67% |
| Austria | 168 | 1.51% |
| Italy | 157 | 1.41% |
| Ireland | 156 | 1.40% |
| Spain | 117 | 1.05% |
| Hungary | 117 | 1.05% |
| Belgium | 89 | 0.80% |
| Norway | 73 | 0.66% |
| Estonia | 67 | 0.60% |
| Greece | 42 | 0.38% |
| Slovakia | 38 | 0.34% |
| Malta | 22 | 0.20% |
| Slovenia | 18 | 0.16% |
| Romania | 16 | 0.14% |
| Latvia | 11 | 0.10% |
| Iceland | 9 | 0.08% |
| Bulgaria | 3 | 0.03% |
| Lithuania | 2 | 0.02% |
| Luxemburg | 2 | 0.02% |
| Grand Total | 11132 | 100.00% |

*This concerns also products approved prior to 2006 via the MRP
